# The Potential Influence of Associated Antidepressants on the Pharmacokinetic Profile of Esketamine in Patients Affected by Treatment-resistant Depression

**DOI:** 10.2174/011570159X356952241216172603

**Published:** 2025-04-07

**Authors:** Marika Alborghetti, Luana Lionetto, Ginevra Lombardozzi, Luca Montaguti, Giada Trovini, Daniela Donato, Giuseppe Costanzi, Donatella De Bernardini, Federica Catapano, Michele Surano, Ilaria Pagano, Alessia Ceccherelli, Edoardo Bianchini, Giorgio Di Lorenzo, Maurizio Simmaco, Giovanni Martinotti, Georgios D. Kotzalidis, Ferdinando Nicoletti, Sergio De Filippis

**Affiliations:** 1 Department of Neuroscience, Mental Health and Sensory Organs, Faculty of Medicine and Psychology, Sapienza University of Rome, Rome, Italy;; 2 Clinical Biochemistry, Mass Spectrometry Section, Sant'Andrea University Hospital, Rome, Italy;; 3 Villa Von Siebenthal Neuropsychiatric Hospital and Clinic, Genzano di Roma, Roma, Italy;; 4 Department of Physiology and Pharmacology, Sapienza University, Rome, Italy;; 5 AGEIS, Université Grenoble Alpes, 38000 Grenoble, France;; 6 Department of Systems Medicine, Tor Vergata University of Rome, Rome, Italy;; 7 IRCCS Fondazione Santa Lucia, Rome, Italy;; 8 Department of Neurosciences, Imaging and Clinical Sciences, Università degli Studi G. D'Annunzio, Chieti, Italy;; 9 Psychopharmacology, Drug Misuse and Novel Psychoactive Substances Research Unit, School of Life and Medical Sciences, University of Hertfordshire, Hatfield AL10 9AB, UK;; 10 IRCCS Neuromed, Pozzilli, Italy

**Keywords:** Esketamine, depression, treatment-resistant, antidepressant drugs, pharmacokinetics, cytochrome P_450_ isoenzymes

## Abstract

**Introduction/Objective:**

Esketamine is administered intranasally in combination with at least another antidepressant in patients with treatment-resistant depression. Some of these antidepressants might affect ketamine’s pharmacokinetic profile by inhibiting cytochrome-P_450_ (CYP_450_) isoforms. Our aim was to establish how different types of combined antidepressants affect serum and salivary levels of esketamine at the time of maximum plasma concentrations and afterward in TRD patients receiving esketamine in a real-world context.

**Methods:**

Serum and salivary samples were collected from 53 patients receiving intranasal esketamine (56 mg) at baseline, after 20 min (roughly corresponding to *T*_max_), 7 hours (corresponding to the t_½_ value), 24, and 72 hours. Patients were stratified according to the combined antidepressant medication.

**Results:**

Salivary esketamine levels were several-fold higher than the corresponding serum levels at all time points, and showed high inter-individual variability. Serum 20-min post-esketamine levels and AUC_0-72_ levels were significantly higher in patients on antidepressants known to inhibit different isoforms of CY_P450_ (paroxetine, fluoxetine, duloxetine, venlafaxine), with respect to levels detected in patients on sertraline, citalopram, escitalopram, vortioxetine. These changes in the pharmacokinetic profile of esketamine did not affect the clinical outcome of esketamine. However, changes in systolic blood pressure in response to esketamine positively correlated with serum esketamine levels, suggesting a reduction of esketamine dose in patients with cardiovascular comorbidity under treatment with paroxetine, fluoxetine, duloxetine, venlafaxine.

**Conclusion:**

The CYP_450_-related status of co-administered antidepressants may affect esketamine levels. However, the small sample sizes of the co-administered drug subgroups and multiple prescriptions do not allow for drawing strong conclusions.

## 1 INTRODUCTION

The approval of intranasal esketamine by the US Food and Drug Administration (FDA) in 2019 [1] has been a major breakthrough in the therapy of treatment-resistant depression (TRD) [2-7]. Esketamine, the (S)-isomer of ketamine, is a slow N-methyl-D-aspartate (NMDA) receptor channel blocker with high affinity for NMDA receptors containing the GluN2D subunit, which are highly expressed by GABAergic interneurons in the forebrain [8]. NMDA receptor blockade by esketamine restrains the activity of parvalbumin-positive (PV^+^) and other types of GABAergic neurons in the cerebral cortex and hippocampus, resulting in disinhibition of pyramidal neurons and enhanced glutamate release at excitatory synapses. This triggers a chain of reactions, possibly leading to a rapid and sustained antidepressant effect [8]. Similarly to psilocybin, the therapeutic effects of esketamine may go beyond its acute administration [9], despite discontinuation may decrease its relapse-prevention effect [10]. An increased formation of dendritic spines mediates the long-lasting effect of ketamine on depressive-like behavior in preclinical studies [11], although the exact mechanisms of its action are not fully elucidated and may involve additional paths [12].

In patients with TRD (*i.e*., patients who do not respond to at least two antidepressants taken for adequate time intervals), esketamine is administered intranasally with long inter-dose intervals (initially twice a week, down to once every two weeks) at doses of 28, 56 or 84 mg, always in add-on with other antidepressants (either a Selective Serotonin Reuptake Inhibitor (SSRI) or a Serotonin-Norepinephrine Reuptake Inhibitor (SNRI) in the EU and any antidepressant in the US) [13]. Given the multitude of possible drug combinations, the possibility to obtain sufficiently powered co-administered drug subgroups is seriously hampered. The occurrence of potential adverse events, such as dizziness, sedation, dissociation, confusion, and increased blood pressure [3], is a limitation to the use of esketamine, although, in most cases, these adverse effects are mild to moderate. Some of these adverse events are directly related to serum esketamine levels, and it is therefore important to disclose the extrinsic and intrinsic factors that influence the pharmacokinetic (PK) profile of esketamine in order to predict potential negative outcomes in the real world.

After intranasal administration, esketamine is rapidly absorbed and the time to reach maximum plasma concentrations (*T*_max_) is 20-40 minutes, with an elimination half-life (t_½_) of 7-12 hours [14]. Esketamine undergoes hepatic metabolism and is demethylated into the inactive compound, noresketamine, by the P450 cytochrome (CYP_450_) isoenzymes CYP2B6 and CYP3A4, and to a lesser extent, CYP2C9 and CYP2C19 [14-17]. Noresketamine is also metabolized by CYP isoforms, and final metabolites undergo glucuronidation [16]; https://www.accessdata.fda.gov/drugsatfda_docs/label/2020/211243s003lbl.pdf. Co-treatment with ticlopidine or clarithromycine, which inhibit CYP2B6 and CYP3A4, respectively, have a negligible effect on the PK profile of esketamine (Data on File. Esketamine. Summary of Clinical Pharmacology. Janssen Research & Development, LLC. EMDS-ERI-149761559; 2018). This might reflect the redundant function of different CYP isoforms in esketamine metabolism.

A PK interaction cannot be excluded with SSRI and SNRI antidepressants which inhibit multiple CYP isoforms. For example, paroxetine is the strongest CYP2B6 inhibitor of all antidepressants [18], fluoxetine inhibits multiple isoforms of cytochrome-P_450_, such as CYP2B6, CYP2C9, CYP2C19, and CYP2D6 [19-21], duloxetine inhibits CYP2B6, CYP2D6 and CYP3A4 [22-24], while venlafaxine inhibits CYP2D6; in contrast, its metabolite, desvenlafaxine, is a weak inhibitor of CYP2D6 and CYP3A4 [22, 25]. Other antidepressants, such as the SSRIs sertraline, citalopram, and escitalopram, and the multimodal antidepressant, vortioxetine, have small to negligible effects on the drug’s metabolism.

Here, we have measured serum and salivary esketamine levels at different times after intranasal administration of 56 mg of esketamine in patients under treatment with different types of antidepressants, arbitrarily grouped on the basis of the expected impact (based on the results of the above-mentioned literature) of the different antidepressants on esketamine metabolism. We hypothesized that esketamine levels would be affected by co-administered drug type according to its CYP450 binding characteristics. In particular, we expected that inhibitors of the CYP2B6 and CYP3A4 isoenzymes would increase esketamine blood levels and that this could affect in turn its clinical effect and/or adverse effects. Our aim was to see whether the pharmacokinetics of intranasal esketamine are affected by the pharmacokinetics of the co-administered drug(s) and how this related to the clinical effects of esketamine and the emergence of adverse events.

## 2 MATERIALS AND METHODS

### 2.1 Study Population

We recruited 53 adult patients affected by TRD who were candidates to receive esketamine 56 mg intranasally (median age, 51.9 years; range from 20 to 73 years). People with the following conditions were excluded from the study: neurological conditions of inflammatory, neurodegenerative, or comitial nature; patients with autism spectrum disorders; endocrinological conditions; patients currently treated with anti-inflammatory, immunosuppressant/immunomodulator drugs; those with active infectious/inflammatory disease; with a history of cancer, hematological disorders, chronic obstructive pulmonary disease (COPD) or asthma, kidney or liver failure, cardiovascular disease (coronary syndromes, cardiomyopathy, or heart failure), autoimmune diseases; history of traumatic brain injury within the past year, and failure to provide free, informed consent. The study conformed to the Principles of Human Rights, as adopted by the World Medical Association at the 18^th^ WMA General Assembly in Helsinki, Finland, in June 1964 and subsequently amended at the 64^th^ WMA General Assembly in Fortaleza, Ceará, Brazil, in October 2013. The study received approval from the local Ethics Committee (protocol N. 0011318/203, 19 JAN 2023).

Patients were stratified according to the antidepressant therapy they used in combination with esketamine: (i) SSRIs that inhibit CYP2B6 or CYP3A4 (paroxetine, fluoxetine); (ii) duloxetine; (iii) venlafaxine/desvenlafaxine; (iv) SSRIs that do not inhibit CYP2B6 or CYP3A4 significantly (sertraline, citalopram, escitalopram); (v) vortioxetine; and (vi) other antidepressants (clomipramine, trazodone, and mirtazapine).

Blood samples and saliva were collected at the following times: baseline (BL), *i.e*., before receiving intranasal esketamine; 20 minutes after intranasal esketamine administration, approximately corresponding to the *T*_max_ value of intranasal esketamine; 7 hours after intranasal esketamine administration, corresponding approximately to the T_½_ value of intranasal esketamine; 24 hours after intranasal esketamine administration; and 72 hours after intranasal esketamine administration, *i.e*. before the next esketamine administration.

Adverse events of intranasal administration of esketamine were evaluated 20 min post-esketamine initiation, such as changes in systolic and diastolic blood pressure, vertigo, dissociation, dizziness, sedation, headache, nausea and vomiting, and confusion.

To assess the changes in clinical status with treatment, we administered the following clinical scales at BL and one month after the first intranasal esketamine: clinician-rated Brief Psychiatric Rating Scale-version 4.0 (BPRS-24) to evaluate psychiatric symptoms [26,27]; Montgomery-Åsberg Depression Rating Scale (MADRS) [28] and Hamilton Depression Rating Scale (HAM-D) [29] to assess depression; Hamilton Anxiety Rating Scale (HAM-A) [30] to assess anxiety; Young Mania Rating Scale (YMRS) [31] to assess manic symptoms, Clinical Global Impressions Scale (CGI) [32] to assess the overall severity (CGI-S) and improvement (CGI-I), with CGI-S administered at both timepoints and CGI-I only at 1 month. Self-rated scales included the Beck Depression Inventory, 2^nd^ edition (BDI-II) [33], used to assess the cognitive aspects of depression (Beck’s cognitive triad) [34], and the World Health Organization Quality of Life Scale-Brief (WHOQOL-BREF) to assess patient’s quality-of-life (QoL) [35]. For all scales save the WHOQOL-BREF, higher scores indicate higher psychopathology, while for the WHOQOL-BREF, higher scores reflect better QoL. Furthermore, we administered the clinician-rated Agitation-Calmness Evaluation Scale (ACES) [36] to assess agitation *vs.* sedation during the first esketamine administration phase (BL to 72 hours). ACES is a single-item scale, in which scores from 1 to 3 are inversely related to the degree of agitation, and scores from 5 to 9 are directly related to calmness and sedation, until the score of 8 and 9, which indicate deep sleep and unarousable state, respectively. Finally, we evaluated dissociation with the Clinician-Administered Dissociative States Scale (CADSS) [37] following the first esketamine administration (BL to 72 hours).

### 2.2 Pharmacokinetic Measurements of Esketamine in Serum and Saliva were Performed using Liquid Chromatography Tandem Mass Spectrometry (LC-MS/MS)

#### 2.2.1 Chemicals and Reagents

A pure compound of S-ketamine was purchased from Merck (St Louis, MO), and internal standard (IS) Ketamine-D_4_ was purchased by Cerilliant (Round Rock, TX). HPLC-grade acetonitrile was purchased from Carlo Erba reagents (Milan, Italy) and formic acid was from Merck (Darmstadt, Germany). Water was deionized and filtered by means of Milli-Q Plus equipment (Millipore Corporation, Bedford, MA).

#### 2.2.2 Stock Solutions and Working Standard

Stock solutions (1 mg/mL) of S-ketamine were prepared by dissolving the pure analyte in acetonitrile 100%. The working solution was prepared by diluting the stock solution with deionized water to obtain a final concentration of 1000 ng/mL for S-ketamine. The working solution was stored at −20°C until use. The calibration curve for the analysis of S-ketamine was obtained by serial dilution of the highest concentration calibration standard solution (500 ng/mL). Calibrator samples and QC samples were treated exactly as patients’ specimens. Stock solutions of ketamine-D4 HCl (100 µg/mL in methanol) were used as Internal Standard (IS). The IS working solution was prepared by diluting the stock solution with acetonitrile to obtain a final concentration of 2 ng/mL.

#### 2.2.3 Sample Preparation

Saliva samples were centrifuged at 3,500g for 10 min and an aliquot of 500 µL of the upper layer was stored at −20°C until processing. Fifty µl of calibrators, QCs, or patient samples were added to 150 µl of IS working solution for saliva and serum deproteinisation. The samples were mixed for 60 seconds and then centrifuged at 14,000 rpm for 20 minutes. Seventy µL of clean upper layer were directly transferred in an autosampler vial and five µL were injected into the chromatographic system.

#### 2.2.4 Chromatographic Conditions

The HPLC analysis was performed using an Exion Liquid Chromatography System (Sciex, Foster City, CA, USA) which included a binary pump, an auto-sampler, a solvent degasser, a column oven, and a controller. Chromatographic separation was performed using a reversed-phase column (Kynetex^®^ 2.6 µm Biphenyl 100 Å pore size, LC Column 100 x 2.1 mm, Phenomenex, CA, USA) equipped with a security guard precolumn (Phenomenex, Torrance, CA, USA) containing the same packing material. The mobile phase consisted of a solution of 0.1% aqueous formic acid (eluent A) and acetonitrile 100% (eluent B); elution was performed at a flow rate of 0.5 mL/min, using a linear gradient from 0% to 100% eluent B in 1 minute. The oven temperature was set at 60°C. The injection volume was 2 µL, and the total analysis time was 4.5 minutes [38].

#### 2.2.5 Mass Spectrometry Conditions

The mass spectrometry method was performed on a 5500 QTrap system (Sciex, Fos-ter City, CA, USA) equipped with a Turbo Ion Spray source. The detector was set in the positive ion mode. The ion spray voltage was set at 5,000 V and the source temperature was 400°C. The collision activation dissociation gas was set at medium value and nitrogen was used as collision gas. The Q1 and Q3 quadrupoles were tuned for the unit mass resolution. The transitions of the precursor ions to the product ions were monitored with a dwell time of 100 ms and 150 ms for S-ketamine and IS, respectively. The instrument was set in the multiple reaction monitoring mode. For each analyte, three transitions were selected: the most intense as quantifiers and the less intense as qualifiers. Mass spectrometer parameters were optimized to maximize sensitivity for all analytes (Table **1**). Data were acquired and processed with Analyst 1.5.1 software.

#### 2.2.6 Method Validation

Processed calibration standards and QC samples were used to develop the calibration curve for the method validation. The validation was conducted considering selectivity, LLOQ, recovery, accuracy and precision, matrix effects, and stability. This method was validated following the European Medicines Agency Guideline on bioanalytical method validation. Specificity, Matrix Effect, and Carry-Over No interference was observed at the retention times of both compounds. The blank saliva and serum used for this study were free from drugs at the retention times of the analytes. The matrix effect was calculated using the ratio of the analyte area spiked in the blank saliva and serum after sample treatment to the analyte area in a working standard solution. All samples were confirmed not to show CVs over 15%. Carry-over was assessed considering the peak area of each compound in a black sample analyzed after the injection of 10 mg/mL standard solution. The peak areas were found to be lower than 20% of the peak area of the LLOQ sample. The linear regression of the calibration curves for S-ketamine showed regression coefficients >0.998 in both saliva and serum. Accuracy ranged from 90.2 to 103.5% and from 87.9 to 110% for the intraday and interday analysis, respectively. The precision data (%CV) showed that all the concentrations of each QC sample analyzed were better than 10% for S-ketamine over the respective LLOQs. The analytical method used in this study reported a mean recovery higher than 86.2% for S-ketamine. Recovery levels were found to be consistent over their respective calibration range, which indicated that the extraction efficiency is not influenced by the concentration in the ranges analyzed. Long-term stability of the compounds, after 60 days at -80°C, as well as after three freeze (-20°C)/thaw (24°C) cycles, was also confirmed.

### 2.3 Area under the Curve (AUC 0.72) Calculation

The AUC was calculated over 72 hours. AUC 0.72 was determined using the Python programming language and the NumPy library, using the trapezoid rule [39].

### 2.4 Statistical Analyses

After having applied the Shapiro-Wilk test to ascertain the normality of distribution of our sample and the Mauchly sphericity test to assess the validity of the Analysis of Variance (ANOVA), we proceeded by performing parametric tests. We used the ANOVA-1way to compare samples for continuous variables and Fishers Least Significant Difference (LSD) to test differences in nominal variables. We used Grubbs’ test (*alpha*=0.05) to identify statistically significant outliers to exclude them from further analyses. Correlations were sought through Pearson’s *r* correlation coefficient. Besides concentrations, we calculated the area under the curve (AUC) as stated above. For all other calculations, we used the IBM Statistical Package for the Social Sciences (SPSS), version 29.0 (September 2022, Armonk, New York: IBM Corporation).

## 3 RESULTS

### 3.1 Effect of Associated Antidepressants on Esketamine Levels

The demographic characteristics of all patients, along with their co-administered drugs are shown in Supplementary Table **1**. These were collected in their demographic sheet, where age and sex were specified, and resulted from their clinical records. Fifty-three patients affected by TRD (26 men and 27 women) were treated with the starting dose of intranasal spray esketamine (56 mg) in association with other antidepressants (25 with a single antidepressant and 28 with at least two antidepressants). Many patients were additionally treated with other psychotropic drugs (mood stabilizers, antipsychotics, and/or benzodiazepines) and/or drugs for the treatment of non-CNS disorders (Table **S1**).

Patients were arbitrarily divided into six groups depending on the antidepressants used in combination with 56 mg of esketamine. All patients being under treatment with at least one SSRI known to inhibit different isoforms of CYP450 and efflux pumps (fluoxetine or paroxetine) were included in the first group, regardless of the presence of additional antidepressants (8 patients); patients under treatment with either duloxetine (n = 14) or venlafaxine/desvenlafaxine (n = 6), which are also known to inhibit drug metabolism/efflux pump were included in the second and third group, respectively; patients under treatment with an SSRIs with a mild-to-negligible impact on drug metabolism (*i.e*., sertraline, citalopram or escitalopram) were included in the fourth group (11 patients); patients under treatment with vortioxetine either alone or combined with antidepressants other than those included in the previous group were included in the fifth group (7 patients); finally, patients under treatment with either tricyclic antidepressants (TCAs), trazodone, or mirtazapine, alone or in combination, were included in the sixth group (7 patients).

**Fig. (1):**
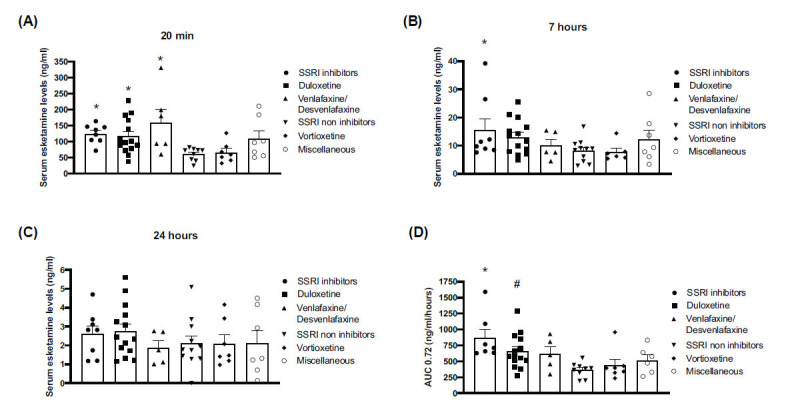
Serum esketamine levels in subgroups of patients under treatment with different types of antidepressants. Patients were arbitrary subdivided into six groups. Group 1 included patients on SSRIs known to inhibit drug metabolism (fluoxetine and paroxetine); groups 2 and 3 included patients who were on duloxetine or venlafaxine/desvenlafaxine, respectively; patients in group 4 were on SSRIs with low/negligible impact on drug metabolism (citalopram/escitalopram/sertraline); patients in group 5 were on vortioxetine; and patients in group 6 were on mirtazapine, trazodone or clomipramine. Esketamine levels (means ± SEM) at 20 minutes, 7 hours, and 24 hours following intranasal esketamine administration are shown in (**A**, **B** and **C**), respectively, AUC 0.72 values (means ± SEM) are shown in (**D**). ONE WAY ANOVA + Fisher’s LSD: A, B, C *p< 0.05 *vs.* groups 4 and 5; (**C**) ^#^*p*<0.05 *vs.* group 4. Statistically significant outliers were identified using Grubbs’ test (*alpha*=0.05) and excluded from further analysis.

Serum esketamine values at 20 min ranged from 25 to >200 ng/mL. Values were significantly higher in patients on paroxetine/fluoxetine (group 1), duloxetine (group 2), or venlafaxine/desvenlafaxine (group 3) with respect to patients on SSRIs with a mild impact on drug metabolism (group 4) and patients treated with vortioxetine (group 5). Values obtained in patients treated with other antidepressants (group 6) were less homogenous and did not differ from values of all other groups (Fig. **1A**). In all groups of patients, esketamine levels progressively decreased at 7 and 24 hours (Figs. **1B**, **C**) and became undetectable after 72 hours (not shown). At 7 hours, there was a significant difference between values obtained in patients treated with fluoxetine/paroxetine and those obtained in patients treated with either SSRIs with mild impact on drug metabolism (group 4) or vortioxetine (group 5) (Fig. **1B**). No significant differences were found at 24 hours (Fig. **1C**).

Similarly, AUC esketamine values were significantly higher in patients treated with fluoxetine/paroxetine *vs.* patients treated with sertraline/citalopram/escitalopram, vortioxetine, or TCAs/mirtazapine/trazodone. Values obtained in patients treated with vortioxetine were also significantly lower than those obtained in patients treated with duloxetine (Fig. **1D**). Of note, one patient of group 1 treated with paroxetine and bupropion (a CYP2D6 inhibitor) was also treated with valproate (CYP2C9, CYP2C19, and CP3A4 inhibitor) and atorvastatine (CYP2B6 inducer, and CYP2C8, CYP2C9, CYP2C19, and CYP2D6 inhibitor) [40]; https://go.drugbank.com. The AUC value of this patient was slightly lower than the average value in group 1 (628.44 *vs.* 867.5 ng/mL/hour). In group 2 (duloxetine), one patient was treated with valproate, three patients with atorvastatin, and one patient with oxcarbazepine (CYP3A4 inducer, CYP2C19 inhibitor). In the latter patient, the AUC value was 46% greater than the average value of the group (954.18 *vs.* 654.87 ng/mL/hour). In group 3 (venlafaxine/desvenlafaxine) one patient was treated with valproate, and another patient with valproate and atorvastatin. The AUC values of both patients were lower than the average value of the group (298 and 598.86 *vs.* 856.78 ng/mL/hour, respectively). In group 4, two patients were treated with valproate and one with topiramate (CYP3A4 inducer, CYP2C19 inhibitor). Interestingly, the AUC values of the patient treated with topiramate were much greater than the average value of the group (921.2 *vs.* 418. 7 ng/mL/hour). One patient of group 5 (vortioxetine) was treated with prednisone (inducer of CYP2B6, CYP2C8, CYP2C9, CYP2C19, CYP3A4, and glycoprotein-P9). The AUC value of this patient was also greater than the average value of the group (957.24 *vs.* 438.53). In group 6 (TCAs/mirtazapine/trazodone), one patient was treated with mirtazapine and oxcarbazepine. The AUC value of this patient was much lower than the average value of the group (259.83 *vs.* 704.86 ng/mL/hour).

**Fig. (2):**
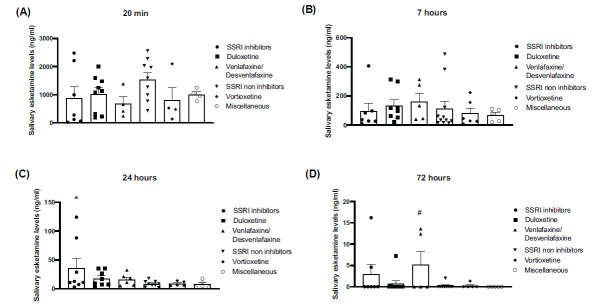
Salivary esketamine levels in subgroups of patients treated with different types of antidepressants. Patients were arbitrary subdivided into six groups. Group 1 included patients on SSRIs known to inhibit drug metabolism (fluoxetine and paroxetine); groups 2 and 3 included patients who were on duloxetine or venlafaxine/desvenlafaxine, respectively; patients in group 4 were on SSRIs with low/negligible impact on drug metabolism (citalopram/escitalopram/sertraline); patients in group 5 were on vortioxetine; and patients in group 6 were on mirtazapine, trazodone or clomipramine. Esketamine levels (means ± SEM) at 20 minutes, 7 hours, and 24 hours following intranasal esketamine administration are shown in **A**, **B** and **C**, respectively, AUC 0.72 values (means ± SEM) are shown in **D**. ONE WAY ANOVA + Fisher’s LSD: **C** *p<0.05 *vs.* groups 4, 5 and 6. **D** ^#^*p*<0.05 *vs.* groups 2, 4, 5 and 6. Statistically significant outliers were identified using Grubbs’ test (alpha = 0.05) and excluded from further analysis.

Interestingly, salivary levels of esketamine at 20 min were several-fold greater than serum levels, with values exceeding 1 μg/mL in 26 patients (Figs. **2A**-**C**). In a few patients treated with fluoxetine/paroxetine, duloxetine, or venlafaxine/desvenlafaxine, esketamine levels were still detectable in the saliva after 72 hours (Fig. **2D**). Because of the high heterogeneity of salivary values, the only significant differences were detected at 24 hours, when values obtained in the fluoxetine/paroxetine group were significantly higher than those obtained in groups 4, 5, and 6 (Fig. **2C**). There was no significant correlation between serum and salivary esketamine values at any timepoint (data not shown).

### 3.2 Therapeutic Efficacy and Adverse Effects in Relation to Serum Esketamine Levels

We evaluated the clinical outcomes and adverse effects of esketamine, regardless of the combined antidepressants, in an attempt to find an association with serum esketamine levels. Adverse effects (changes in blood pressure, vertigo, dissociation, dizziness, sedation, headache, nausea/vomiting, confusion) were evaluated at 20 min, whereas the BPRS, MADRS, HAM-D, HAM-A, BDI, YMRS, WHOQOL-BREF, and CGI clinical scales were administered at BL (*i.e*., prior to the first administration of esketamine), and after 1 month.

**Fig. (3):**
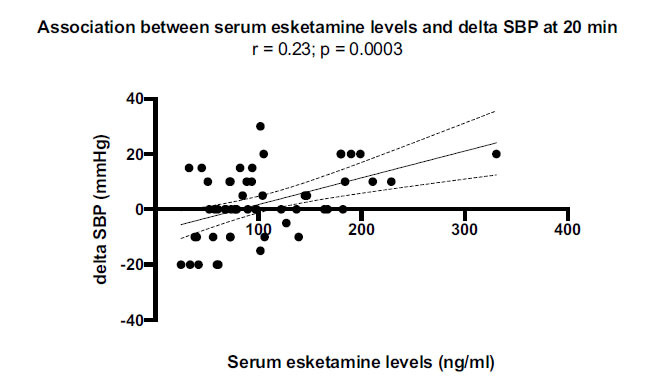
Positive correlation between serum esketamine levels and changes in systolic blood pressure (SBP) at 20 minutes following esketamine administration.

Here, a rise in systolic blood pressure ranging from 5 to 30 mmHg was observed in 23 patients (43.4%), whereas blood pressure was relatively unchanged in 15 patients (28.3%) and reduced in 15 patients (28.3%). There was a significant positive correlation between serum esketamine values and changes in systolic blood pressure at 20 min (*r* = 0.23; *p* = 0.0003) (Fig. **3**). Dizziness (*χ^2^* = 4.114, *p* = 0.043) and confusion (*χ*^2^ = 3.830, *p* < 0.05) were also positively correlated with serum esketamine levels at 20 min post-administration.

**Fig. (4):**
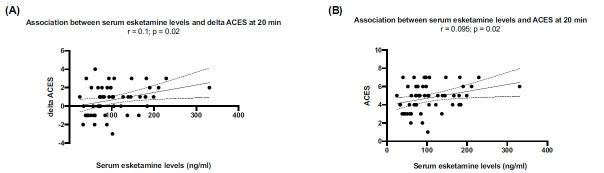
Positive correlation between serum esketamine levels and changes in agitation/calmness scale (ACES) score at 20 minutes following esketamine administration. Correlation with differential ACES (**A**) and absolute ACES scores (**B**) between BL and 20 minutes post intranasal esketamine.

There was a positive correlation between esketamine levels and changes in ACES at 20 minutes (*r* = 0.1; *p* = 0.02) (Figs. **4A** and **B**). All other adverse effects, including dissociation (as assessed with the CADSS) did not correlate with esketamine levels at 20 min (not shown). Of note, most of the adverse events related to esketamine administration in our study resulted in transient, self-limiting, and mild-moderate severity, and had no impact on therapy continuation.

**Fig. (5):**
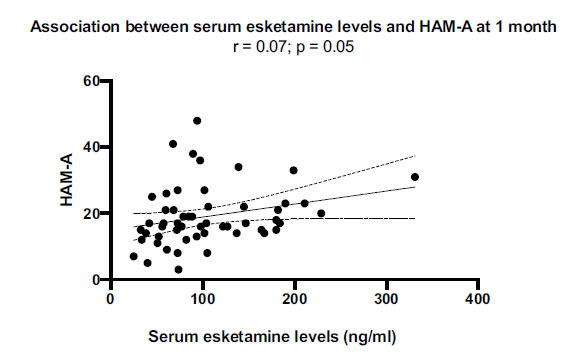
HAM-A scores. Positive correlation between HAM-A scores and esketamine values at 20 minutes and AUC 0.72 values.

Interestingly, scores on the HAM-A, YMRS, and “activity” items of BPRS correlated with esketamine levels at 20 min and/or AUC esketamine values. Esketamine was efficacious in improving anxiety in nearly all patients (HAM-A values dropped in 51 of 53 patients). There was no correlation between esketamine levels measured at *T*_max_ (20 min) and HAM-A score changes from baseline and 1 month (Fig. **S1A**, **B**). However, patients with lower serum levels of esketamine at *T*_max_ resulted in lower scores at HAM-A (Fig. **5**).

**Fig. (6):**
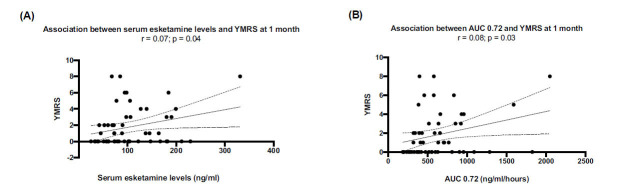
YMRS scores. Positive correlation between YMRS scores and serum esketamine levels (**A**) and AUC 0.72 values (**B**).

One month following initial esketamine administration, YMRS scores decreased in 23 patients (43.4%), remained unchanged in 15 patients (28.3%), and increased in 15 patients (28.3%). Similarly to HAM-A, at *T*_max_ (20 min.) There was a positive correlation between the absolute YMRS score and serum esketamine levels and AUC values, although YMRS values were always <8 (Figs. **6A** and **B**). Changes in YMRS scores between baseline and 1-month after the first esketamine administration did not correlate (Figs. **S2A**, **B**).

**Fig. (7):**
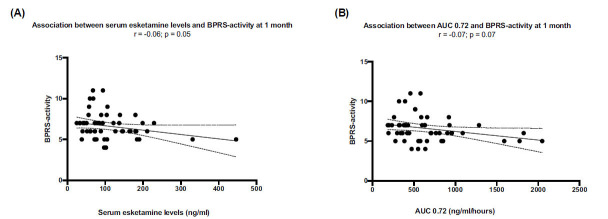
Combination of activity items of BPRS score: 7 (elated mood), 19 (tension), 21 (excitement), 23 (motor hyperactivity), and 24 (mannerism and posturing). Negative correlation between BPRS-activity scores and either esketamine values at 20 minute or AUC 0.72 values are shown in (**A** and **B**), respectively.

A negative correlation was found between serum esketamine level at 20 min or AUC esketamine values and absolute values of the combined 7 (elated mood), 19 (tension), 21 (excitement), 23 (motor hyperactivity), and 24 (mannerism and posturing) activity items of BPRS, meaning that patients with higher exposure to esketamine show a better performance on this item cluster (Fig. **7**).

At 1 month after esketamine administration the global scores of all clinical scales used in this observational study (BPRS-Total, MADRS, BDI, WHOQOL-BREF or CGI) were improved. (Figs. **S3** and **S4**). However, there was no correlation between serum esketamine levels at 20 min or AUC values and scores of all these scales.

## 4 DISCUSSION

Esketamine has been approved worldwide for the add-on treatment of patients affected by TRD, *i.e*., patients who failed to respond to at least two antidepressants. This has significantly expanded the therapeutic options for TRD, which accounts for approximately one-third of all cases of MDD. However, esketamine must be combined with at least another antidepressant (an SSRI/SNRI in the EU; any antidepressant in the US), which, in principle, might interfere with the PK and pharmacodynamics profile of esketamine. Prototypical inhibitors of specific isoforms of CYP_450_ (*i.e*., ticlopidine and clarithromycine) have small effects on the PK profile of esketamine (Data on File. Esketamine. Summary of Clinical Pharmacology. Janssen Research & Development, LLC. EMDS-ERI-149761559; 2018). However, many antidepressants inhibit multiple isoforms of CYP_450_ and might influence esketamine exposure. We found that esketamine exposure was greater when the drug was used in combination with an SNRI (duloxetine, venlafaxine, desvenlafaxine) or with an SSRI known to inhibit drug metabolism (fluoxetine or paroxetine), and lower when esketamine was used in combination to other SSRIs (citalopram, escitalopram, sertraline) or vortioxetine.

Salivary tests revealed that esketamine levels were extremely high and highly variable with no significant correlation with serum values. This suggests that esketamine accumulates in the salivary glands and/or oral cavity after intranasal administration, and the kinetics of the drug in the saliva is critically influenced by a number of unpredictable variables. Thus, we consider measurements of salivary samples to be useless in studying the PK profile of esketamine after intranasal administration.

Although we found that some antidepressants may enhance C_max_ and AUC serum levels of esketamine, these changes did not affect the therapeutic efficacy of esketamine at 1 month. There was no correlation between C_max_ serum levels or AUC values and the extent of clinical improvement evaluated with most clinical scales administered to our patients. Although we found correlations between esketamine levels and absolute values of HAM-A, YMRS, and the sum of 7, 19, 21, 23, and 24 items of BPRS, at 1 month, no correlations were found with D values of the three scales (*i.e*., the difference between 1-month and baseline values). Thus, we cannot conclude that the effect on anxiety or agitation signals detected at 1 month is actually influenced by the kind of antidepressant combined with esketamine as a result of PK/pharmacodynamic interactions.

An intriguing data observed at 20 minutes was a positive correlation between serum esketamine levels and systolic blood pressure. Even if the increase in blood pressure in response to esketamine is known to be generally characterized by transient nature and mild severity [3, 41-43], this aspect could be clinically relevant in special populations. A rise in blood pressure is one of the expected adverse effects of esketamine, even if in phase 3 studies it was observed in< 10% of patients, was mild in severity in most cases, and did not cause treatment interruption [3]. In our study, systolic blood pressure changes were not unidirectional, similar to what occurred in other studies [3, 41-43], despite blood pressure correlated with esketamine blood levels. Our impression was that blood pressure changes were not of clinical significance, and this aligns with what has been observed by others in patients with depression [44, 45]. Thus, based on our results, we can infer that association with drugs that are not supposed to increase peak serum levels of esketamine - such as sertraline, citalopram, escitalopram, or vortioxetine - could be a safer choice in patients with an unstable or clinically significant cardiovascular condition, in order to strongly minimize the risk of an increase in blood pressure.

We also found a positive correlation between peak esketamine levels and the occurrence of dizziness or confusion at 20 min, but, interestingly, there was no association between esketamine levels and psychotomimetic effects evaluated with the CADSS scale. Thus, both the occurrence and severity of dissociative symptoms in response to esketamine appeared to be unrelated to serum drug levels. Based on these results, we can assume that the PK interaction between esketamine and associated antidepressant(s) is not relevant to the occurrence and severity of esketamine-induced dissociative symptoms.

Finally, we observed that serum esketamine levels at 20 min positively correlated with the ACES score, indicating a greater extent of sedation with higher esketamine exposure. This finding may have relevance for the choice of the associated antidepressant in old patients or in patients who are under treatment with benzodiazepines or other CNS depressants. As outlined in the Introduction, esketamine inhibits NMDA receptors expressed by GABAergic interneurons, thereby restraining inhibition at synapses between interneurons and pyramidal neurons [8]. The increased sedation associated with higher esketamine levels might be caused by a dose-dependent inhibition of NMDA receptors expressed by pyramidal neurons in the cerebral cortex. This hypothesis warrants further investigation.

In our study, we observed a lack of correlation between esketamine levels and changes (improvement) in scores of anxiety, general psychopathology, and mania rating scales. This would suggest that esketamine is not active in these dimensions, but it is specific to depression. However, the esketamine levels and AUC did correlate with absolute levels of mania and anxiety and missed by little the correlation with general psychopathology levels. We have no explanation to provide for this.

A major limitation of the study was the lack of information on genetic variants of CYP_450_ involved in esketamine metabolism, and the presence of additional drugs, such as valproate, atorvastatine, oxcarbazepine, or prednisone, which might have influenced esketamine metabolism and/or elimination, confounding the interpretation of our findings. In spite of these limitations, our data suggest that the PK profile of esketamine is influenced by the associated antidepressants, but fluctuations of serum esketamine levels have no significant impact on clinical improvement one month after the initial administration of 56 mg esketamine. This aspect is particularly relevant because, in the final instance, offers physicians the opportunity to move towards a wide range of potential esketamine-antidepressant combinations, based on specific patients' needs.

In contrast, the increase in blood pressure, and the occurrence of dizziness and confusion, which are mild and transient adverse effects reported after intranasal esketamine, might be related to drug exposure, and, therefore, influenced by the nature of the associated antidepressants. This might contribute (in addition to the pharmacodynamics profile) to the choice of the safest antidepressant in particular sub-cohorts of TRD patients, *e.g*., in patients with cardiovascular comorbidity.

Limitations of our study include small sample sizes and multiple antidepressants used, which prevent us from drawing firm conclusions. In particular, the small sample size prevented us from reaching adequately powered groups of individual drugs to carry out differential analyses. Furthermore, we did not assess cognition in this study. This could have been relevant, as esketamine has been shown to impair verbal learning and memory in healthy people [46] and in view of the fact that one of the drugs used here in some cases, vortioxetine, has shown enhancing effects on cognition [47, 48]. Moreover, we did not assess the pharmacogenomics of our patients due to the difficulties of obtaining ethical approval for such testing. Our study was conducted on a European white-only population, hence our data cannot extend to other populations, such as African-Black or Asian populations, who were shown to differ in their CYP450 isoenzyme variants [49-51]. Finally, the discrepancy between salivary and blood concentrations is puzzling and not easy to explain. Further studies are required to see whether this inconsistency is widespread or belongs only to our study and to investigate its possible underpinnings.

## CONCLUSION

Our data suggest that the PK profile of the antidepressant(s) combined with esketamine might influence esketamine levels and its resulting adverse effects detected at a time corresponding to its *T*_max_ value, although the use of two or more drugs in combination with esketamine is a clear limitation of our study and does not allow us to draw sound conclusions. In contrast, the short-term clinical outcome evaluated after 1 month did not appear to be influenced by esketamine exposure at least under our experimental conditions (all patients had been treated with 56 mg of esketamine regardless of their previous treatment with one or more antidepressants, sometimes combined with mood stabilizers or benzodiazepines). Our data suggest that the PK profile of the antidepressants combined with esketamine should be taken into consideration in optimizing the safety profile of intranasal esketamine in patients with TRD. However, caution is needed in interpreting our results due to the small size of the co-administered drug groups that hinder statistical analysis, thus preventing us from drawing definitive conclusions.

## Figures and Tables

**Table 1:** Mass spectrometer parameters.

**Analyte**	**Precursor Ion (*m/z*)**	**Fragment (m/z)**	**DP (V)**	**EP (V)**	**CE (V)**	**CXP (V)**
S-ketamine 1	238.000	219.800	150	10.0	21.900	15.2
S-ketamine 2	238.000	207.000	150	10.0	25.000	11.1
S-ketamine 3	238.000	125.200	150	10.0	30.000	13.0
Ketamine-D_4 _1	240.200	224.100	100	10.0	25.000	13.0
Ketamine-D_4 _2	240.200	211.200	100	10.0	25.000	13.0
Ketamine-D_4 _3	240.200	129.200	100	10.0	40.000	13.0

## Data Availability

Data will be made available upon reasonable request to the corresponding author.

## LIST OF ABBREVIATIONS

ACES: Clinician-rated Agitation-calmness Evaluation Scale
AUC: Area Under The Curve
BDI: Beck Depression Inventory
BPRS: Brief Psychiatric Rating Scale
CADSS: Clinician-administered Dissociative States Scale
CGI: Clinical Global Impressions scale
CNS: Central Nervous System
CYP: Cytochrome
PEU: European Union
GABA: Gamma-amino Butyric Acid
HAM-A: Hamilton Anxiety Rating Scale
MADRS: Montgomery-åsberg Depression Rating Scale
NMDA: *N*-methyl-D-aspartate
PK: Pharmacokinetic/Pharmacokinetics
QoL: Quality of Life
SNRI: Serotonin-noradrenaline Reuptake Inhibitor(s)
SSRI: Selective Serotonin Reuptake Inhibitor(s)
t_½_: Halftime
*T*_max_: The time at which plasma concentrations of a given substance is at its highest levels
TRD: Treatment-resistant Depression
US: United States (of America)
WHOQOL-BREF: World Health Organization Quality-of-Life scale, Brief
WMA: World Medical Association
YMRS: Young Mania Rating Scale
