# Supplementary material for: The Potential Influence of Associated Antidepressants on the Pharmacokinetic Profile of Esketamine in Patients Affected by Treatment-resistant Depression
Source: Curr Neuropharmacol. 2025 Apr 7;23(10):1301–12. doi: 10.2174/011570159X356952241216172603 (PMC12307992; doi:10.2174/011570159X356952241216172603)
Supplement: Supplementary file 1 [file CN-23-10-1301_SD1.pdf]

Supplementary Material

The Potential Influence of Associated Antidepressants on the Pharmacokinetic Profile of Esketamine in Patients Affected by Treatment-resistant Depression

Marika Alborghetti[1,*,#], Luana Lionetto[2,#], Ginevra Lombardozzi[3,#], Luca Montaguti[4,#], Giada Trovini[3], Daniela Donato[3], Giuseppe Costanzi[2], Donatella De Bernardini[2], Federica Catapano[4], Michele Surano[4], Ilaria Pagano[4], Alessia Ceccherelli[4], Edoardo Bianchini[1,5], Giorgio Di Lorenzo[6,7], Maurizio Simmaco[1,2], Giovanni Martinotti[8,9], Georgios D. Kotzalidis[3], Ferdinando Nicoletti[4,10] and Sergio De Filippis[3]

[1]Department of Neuroscience, Mental Health and Sensory Organs, Faculty of Medicine and Psychology, Sapienza University of Rome, Rome, Italy; [2]Clinical Biochemistry, Mass Spectrometry Section, Sant'Andrea University Hospital, Rome, Italy; [3]Villa Von Siebenthal Neuropsychiatric Hospital and Clinic, Genzano di Roma, Italy; [4]Department of Physiology and Pharmacology, Sapienza University, Rome, Italy; [5]AGEIS, Université Grenoble Alpes, 38000 Grenoble, France; [6]Department of Systems Medicine, Tor Vergata University of Rome, Rome, Italy; [7]IRCCS Fondazione Santa Lucia, Rome, Italy; [8]Department of Neurosciences, Imaging and Clinical Sciences, Università degli Studi G. D'Annunzio, Chieti, Italy; [9]Psychopharmacology, Drug Misuse and Novel Psychoactive Substances Research Unit, School of Life and Medical Sciences, University of Hertfordshire, Hatfield AL10 9AB, UK; [10]IRCCS Neuromed, Pozzilli, Italy

1875-6190/25 $65.00+.00 **© 2025 Bentham Science Publishers**

Supplementary Table 1. Use of psychotropic and non-psychotropic drugs by all patients at the time of esketamine treatment.

ID patient	Age, sex	Antidepressants (per day)	Antipsychotic drugs (per day)	Benzodiazepins (per day)	Mood stabilizers agents (per day)	Others medications (per day)
**G.M.**	38, man	vortioxetine 20 mg	quetiapine 250 mg;	lorazepam 2.5 mg;	carbolithium 600 mg; pregabalin 150 mg;	
**A.M***	59, woman	duloxetine 120 mg; trazodone 300 mg	olanzapine 5 mg		lamotrigine 100 mg	levothyroxine 75 mcg
**C.F.***	49, man	paroxetine 35 mg; trazodone 300 mg	quetiapine 800 mg; aripiprazole 400 mg/28 days	lorazepam 2.5 mg; flurazepam 30 mg; alprazolam 1 mg	gabapentin 1600 mg	
**P.B.**	65, woman	clomipramine 150 mg; trazodone 100 mg	brexpiprazole 1 mg	lorazepam 2.5 mg	carbolithium 900 mg	metformin 2500 mg; acetylsalicylic acid 100 mg; bisoprolol 1.25 mg
**S.L.**	50, woman	vortioxetine 20 mg; mirtazapine 30 mg	quetiapine 175 mg	lorazepam 2.5 mg	lithium sulphate 124.5 mg	nebivolol 2.5 mg
**S.R.**	27, woman	clomipramine 75 mg; vortioxetine 10 mg die	brexpiprazole 3 mg	delorazepam 2 mg; zolpidem 10 mg	carbolithium 1200 mg	drospirenone 0.02 mg; pramipexole 0.18 mg
**N.S.**	59, woman	duloxetine 120 mg; mirtazapine 30 mg	olanzapine 10 mg	lorazepam 1 mg	pregabalin 75 mg; carbolithium 150 mg	bisoprolol 1.25 mg; enalapril 20 mg; acetylsalicylic acid 100 mg; metformin 500 mg; pantoprazole 40 mg; atorvastatin 20 mg
**N.M.**	50, man	citalopram 20 mg; duloxetine 60 mg	lurasidone 74 mg		lamotrigine 100 mg	amlodipine 10 mg; atenolol 100 mg; atorvastatin 40 mg
**L.F.**	54, woman	duloxetine 120 mg; vortioxetine 20 mg	brexpiprazole 4 mg	clonazepam 1 mg	gabapentin 1200 mg	lansoprazole 30 mg; estradiol 25 mcg
**P.M.**	49, woman	citalopram 20 mg		prazepam 5 mg; lorazepam 2.5 mg; zolpidem 10 mg	lithium sulphate 124.5 mg	levothyroxine 75 mcg; lansoprazole 30 mg; ethinylestradiol 0.03 mg
**D.C.N**	65, man	vortioxetine 20 mg; sertraline 20 mg	brexpiprazole 2 mg	clonazepam 1.5 mg		telmisartan 40 mg; flecainide 100 mg; sylodosin 8 mg; metoprolol 100 mg; esomeprazole 20 mg
**C.F.**	50, man	vortioxetine 20 mg; sertraline 150 mg		prazepam 5 mg; lorazepam 2,5 mg; zolpidem 10 mg	lithium sulphate 124,5 mg	amlodipine 5 mg
**O.S.**	49, man	sertraline 50 mg die	brexpiprazole 2 mg	delorazepam 4 mg	valproic acid 1000 mg	pramipexole 0.54 mg
**T.G.**	58, man	vortioxetine 20 mg; trazodone 300 mg; clomipramine 75 mg	lurasidone 74 mg	zolpidem 10 mg	carbolithium 600 mg; lamotrigine 100 mg	dutrasteride 0,5 mg; allopurinol 300 mg; lansoprazole 30 mg; ramipril 5 mg; fenofibrate 200 mg
**F.M.**	55, man	vortioxetine 20 mg; duloxetine 120 mg; trazodone 75 mg	quetiapine 350 mg;	delorazepam 2 mg	pregabalin 450 mg	esomeprazole 40 mg
**C.D.**	43, man	escitalopram 20 mg		delorazepam 2 mg		irbesartan 150 mg
**A.R.**	56, woman	paroxetine 20 mg		delorazepam 2.5 mg	lithium sulphate 124.5 mg; gabapentin 300 mg	bisoprolol 2.5 mg
**V.L.**	65, woman	sertraline 100 mg; mirtazapine 30 mg	quetiapine 50 mg	delorazepam 2 mg	carbolithium 300 mg;	nadolol 20 mg; valsartan 80 mg
**I.B.K.**	44, man	sertraline 100 mg	quetiapine 50 mg	lorazepam 2.5 mg		
**D.B.L.**	67, woman	escitalopram 20 mg	quetiapine 200 mg	delorazepam 1 mg	valproic acid 300 mg;	propranolol 40 mg; pantoprazole 40 mg
**D.P.**	53, man	trazodone 75 mg; venlafaxine 75 mg	lurasidone 148 mg	clonazepam 50 gtt	valproic acid 800 mg; gabapentin 800 mg	
**C.A.**	46, man	fluoxetine 20 mg; vortioxetine 20 mg	brexpiprazole 2 mg		lithium sulphate 83 mg	levothyroxine 25 mcg
**C.L.**	35, man	duloxetine 30 mg	lurasidone 37 mg	clonazepam 4 mg	valproic acid 300 mg	
**Z.M.R.**	47, woman	fluoxetine 20 mg	quetiapine 5 mg	lorazepam 2.5 mg	gabapentin 300 mg	
**R.S.**	28, man	duloxetine 80 mg			lithium sulphate 83 mg	
**L.M.P.***	58, woman	escitalopram 10 mg	brexpiprazole 2 mg; quetiapine 50 mg		lamotrigine 200 mg; valproic acid 750 mg	levothyroxine 75 mcg
**M.A.**	58, woman	desvenlafaxine 100 mg	brexpiprazole 4 mg	zolpidem 10 mg; alprazolam 15 mg	lamotrigine 50 mg; lithium sulphate 83 mg	
**S.L.**	51, woman	paroxetine 30 mg	lurasidone 74 mg; quetiapine 50 mg	triazolam 250 mg; lorazepam 10 mg	lithium sulphate 124.5 mg	nebivolol 2.5 mg
**C.E.**	63, woman	trazodone 150 mg	olanzapine 10 mg; brexpiprazole 2 mg	delorazepam 2 mg; flurazepam 30 mg	pregabalin 150 mg	pravastatin 40 mg; bisoprolol 1,25 mg
**M.A.**	49, woman	trazodone 20 mg; duloxetine 60 mg; amitriptyline 25 mg		alprazolam 2 mg	lurasidone 74 mg	bisoprolol 1,25 mg; ramipril 10/25 mg
**C.F. ***	49, man	duloxetine 90 mg	clozapine 200 mg; quetiapine 300 mg	prazepam 30 mg		atorvastatin 40 mg; lansoprazole 30 mg
**L.A.**	73, woman	buproprion 150 mg, paroxetine 15 mg	quetiapine 25 mg		valproic acid 150 mg; gabapentin 75 mg	losartan/idroclorotiazide100/25 mg; atorvastatin 20 mg; metformin 500 mg
**D.L.**	48, woman	mirtazapine 20 mg	olanzapine 5 mg	zolpidem 30 mg	oxcarbazepine 600 mg	
**D.E.**	48, man	sertraline 50 mg		zolpidem 12.5 mg		
**M.F.**	55, man	duloxetine 60 mg; trazodone 150 mg	aripiprazole 400 mg/28 days	clonazepam 2.5 mg	oxcarbazepine 900 mg	
**C.G.**	64, man	venlafaxine 150 mg	olanzapine 10 mg	lorazepam 2,5 mg	valproic acid 1000 mg	atorvastatin 20 mg; pantoprazole 20 mg; telmisartan/hydrochlorothiazide 80/25 mg
**V.L.**	47, woman	escitalopram 20 mg	quetiapine 250 mg		lamotrigine 75 mg	
**V.D.**	21, man	duloxetine 90 mg; trazodone 75 mg; vortioxetine 10 mg	brexpiprazole 1 mg	delorazepam 1 mg	lithium sulphate 83 mg	
**A.M.***	61, woman	duloxetine 120 mg, amitriptyline 35 mg	cariprazine 1.5 mg		pregabalin 375 mg; lamotrigine 100 mg	metformin 750 mg; levothyroxine 75 mcg; cetirizine 10 mg
**P.L.N**	60, woman	vortioxetine 20 mg; clomipramine 75 mg	lurasidone 74 mg		carbolithium 750 mg	
**S.M.**	57, woman	trazodone 135 mg; fluoxetine 30 mg	trifluoperazine 2 mg; quetiapine 50 mg	delorazepam 2 mg		esomeprazole 20 mg; olmesartan 10 mg; cetirizine 20 mg
**M.F.**	62, woman	clomipramine 225 mg		alprazolam 2 mg	lithium sulphate 83 mg	

**T.R.**	57, man	escitalopram 20 mg; venlafaxine 300 mg	olanzapine 5 mg	lorazepam 2.5 mg	carbolithium 600 mg	
**T.C.**	69, man	venlafaxine 300 mg	L-sulpiride 50 mg; quetiapine 400 mg		lithium sulphate 124,5 mg	
**S.R.**	63, man	clomipramine 150 mg; trazodone 150 mg		lorazepam 2.5 mg		
**L.G.**	49, man	clomipramine 150 mg	olanzapine 5 mg		lithium sulphate 124.5 mg	
**G.G.**	47, man	venlafaxine 150 mg	quetiapine 00 mg	zolpidem 10 mg		
**Z.A.A.**	71, man	trazodone 150 mg; mirtazapine 30 mg	brexpiprazole 3 mg; reboxetine 4 mg; olanzapine 10 mg	delorazepam 1 mg		
**C.R.**	48, woman	trazodone 150 mg, duloxetine 60 mg	quetiapine 200 mg	pregabalin 300 mg; delorazepam 1.5 mg; zolpidem 10 mg	lithium sulphate 176 mg	
**A.L**	60, man	vortioxetine 20 mg	olanzapine 7.5 mg	alprazolam 1 mg		prednisone 5 mg; furosemide 50 mg; pantoprazole 80 mg
**M.B.**	20, woman	fluoxetine 40 mg	quetiapine 300 mg; brexpiprazole 20 mg	alprazolam 1 mg; lorazepam 2 mg	pregabalin 175 mg; lithium sulphate 83 mg	
**L.M.P.***	58, woman	citalopram 20 mg	quetiapine 50 mg; brexpiprazole 2 mg		lamotrigine 200 mg; valproic acid 1000 mg	levothyroxine 75 mcg; propranolol 10 mg
**A.M.***	61, woman	duloxetine 120 mg; trazodone 150 mg	brexpiprazole 2 mg	zolpidem 10 mg; lorazepam 2.5 mg	pregabalin 375 mg; lamotrigine 100 mg	levothyroxine 75 mcg

* these patients have switched antidepressant medication. Esketamine levels have been measured twice in patients C.F. and L.M.P., and three times in patient A.M.

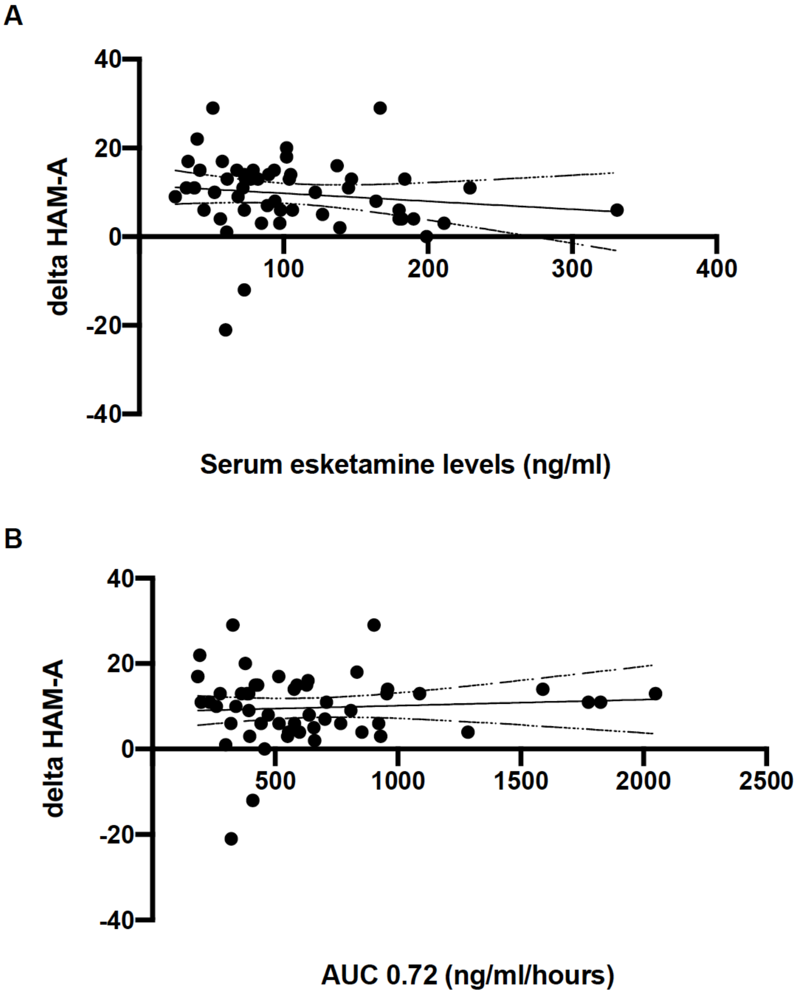

Supplementary Figure 1. Lack of correlation between peak serum esketamine levels (A) and UC0.72 (B) and delta-HAM-A.
Delta scores are expressed as the difference between baseline and 1-month values.

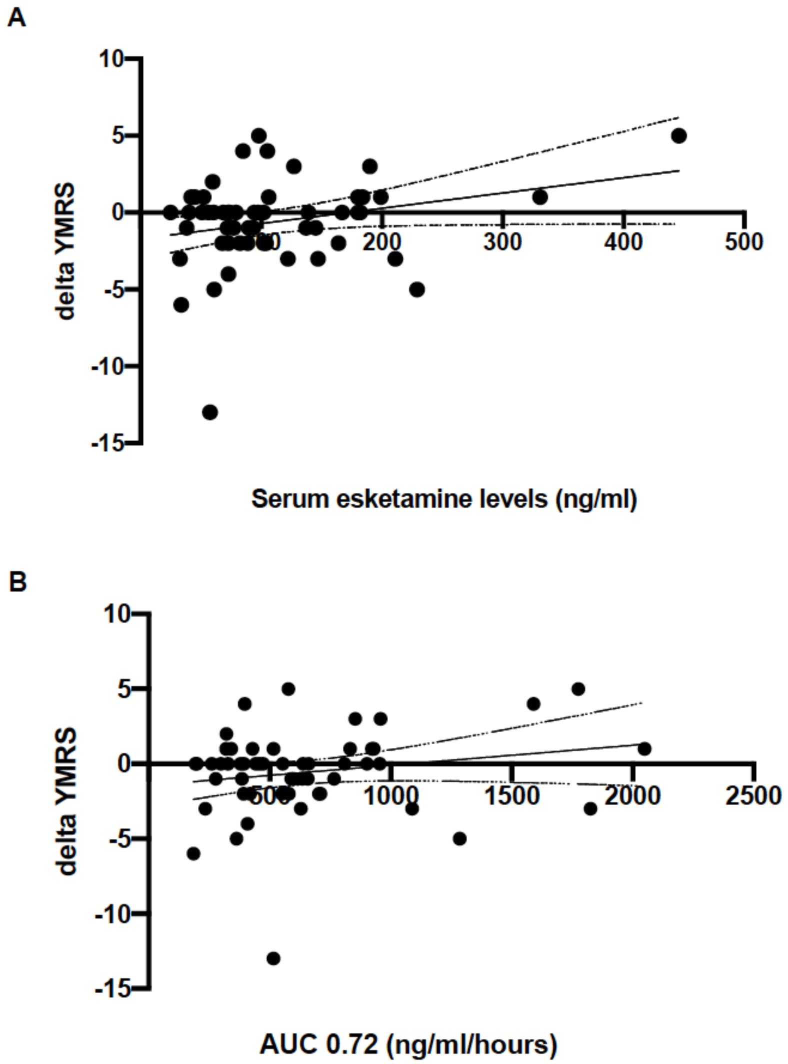

Supplementary Figure 2. Lack of correlation between peak serum esketamine levels (A) and UC0.72 (B) and delta-YMRS.
Delta scores are expressed as the difference between baseline and 1-month values.

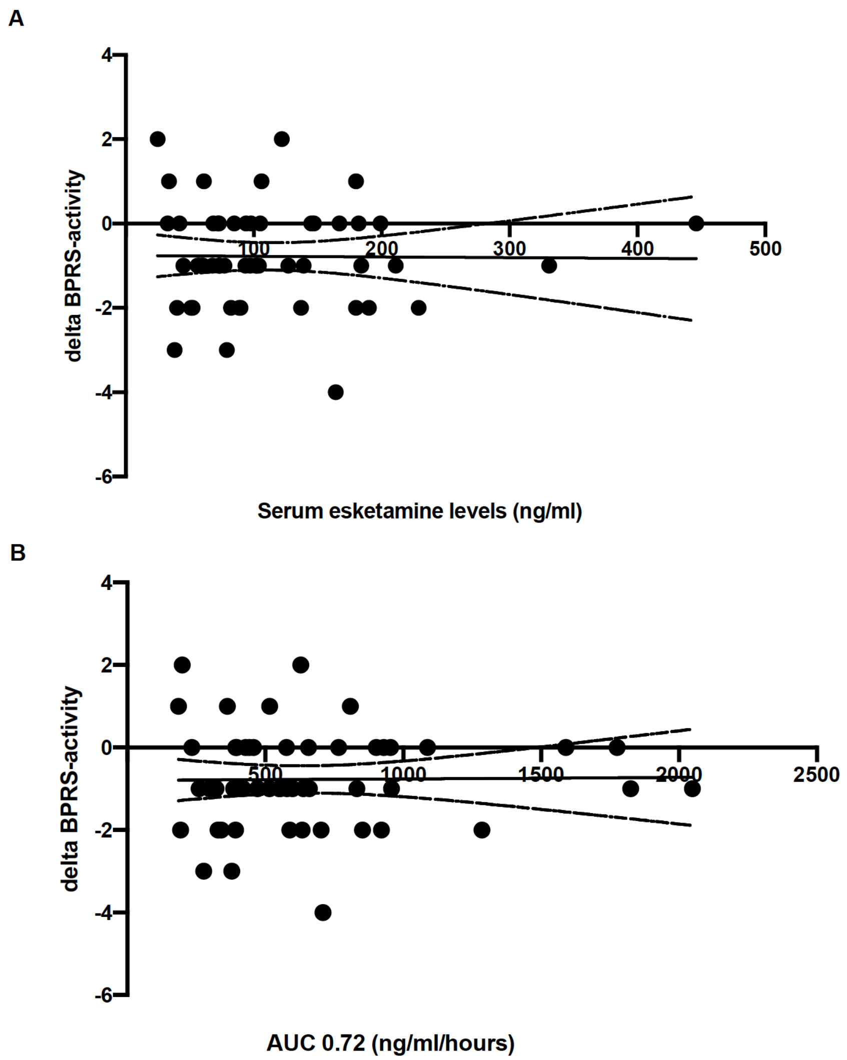

Supplementary Figure 3. Lack of correlation between peak serum esketamine levels (A) and UC0.72 (B) and delta-BPRS-activity subdomains.
Delta scores are expressed as the difference between baseline and 1-month values.

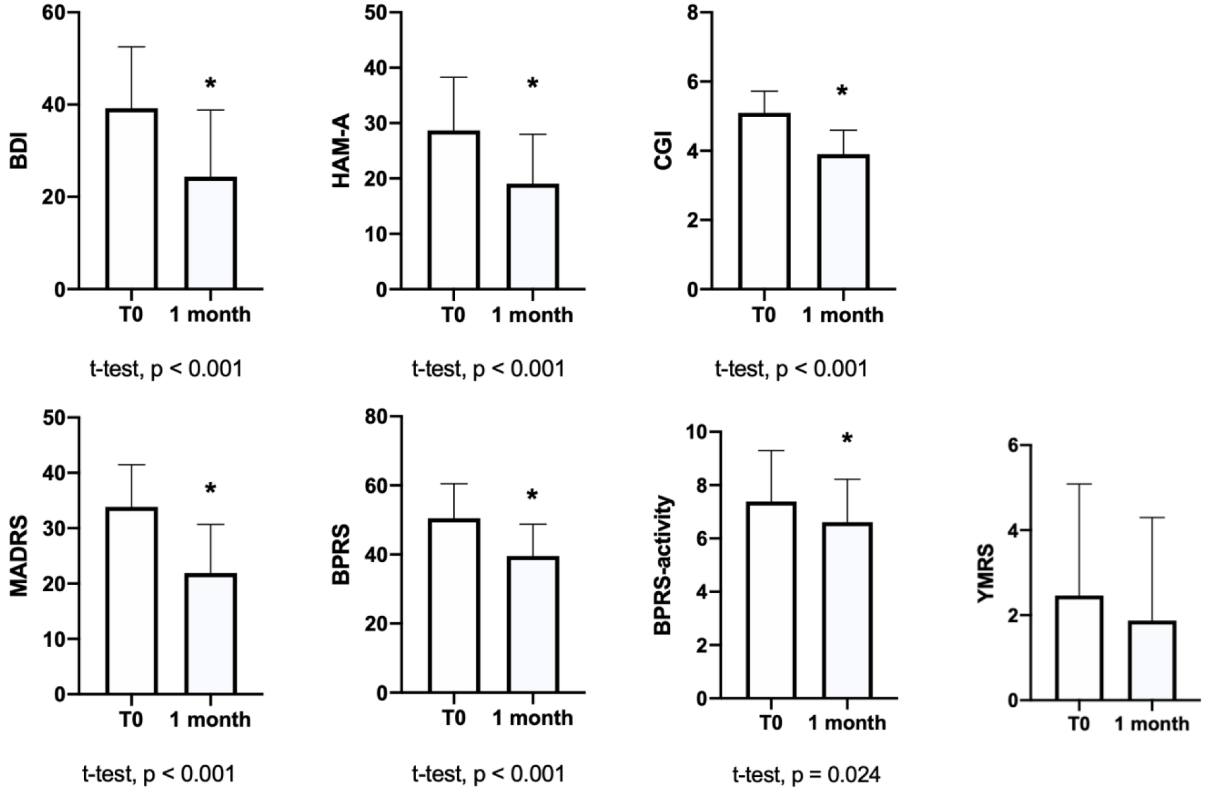

Supplementary Figure 4. Efficacy of esketamine in improving clinical symptoms in our cohort of patients affected by treatment-resistant depression.
BDI: Beck Depression Inventory; HAM-A: Hamilton Anxiety Scale; CGI: Clinical Global Impression; MADRS: Montgomery-Åsberg Depression Rating Scale; BPRS: Brief Psychiatric Rating Scale; YMRS: Young Mania Rating Scale.
T-test (mean values + SD). **p*<0.05 vs baseline values (T0).
